# The Role of Pancreatic Stone Protein in Diagnosis of Early Onset Neonatal Sepsis

**DOI:** 10.1155/2016/1035856

**Published:** 2016-09-05

**Authors:** Anwar A. Rass, Mohamed A. Talat, Mohamed A. Arafa, Hosam F. El-Saadany, Ezzat K. Amin, Mohamed Mohamed Abdelsalam, Mona A. Mansour, Naglaa A. Khalifa, Lamiaa Mahmoud Kamel

**Affiliations:** ^1^Department of Pediatrics, Faculty of Medicine, Zagazig University, Zagazig 44519, Egypt; ^2^Ministry of Health, Zagazig, Egypt; ^3^Clinical Pathology, Faculty of Medicine, Zagazig University, Zagazig 44519, Egypt

## Abstract

*Introduction*. Early diagnosis and treatment of neonatal sepsis may help decrease neonatal mortality.* Aim of the Study*. To evaluate the role of pancreatic stone protein as a marker for early onset neonatal sepsis.* Methods*. A hospital-based prospective study was conducted on 104 (52 uninfected and 52 infected neonates) admitted to the Neonatal Intensive Care Unit (NICU) of Zagazig University hospitals during the period from April 2014 to April 2015. All newborns were subjected to full history taking, careful neonatal assessment, blood, C-reactive protein (CRP), and serum pancreatic stone protein.* Results*. Serum PSP levels were significantly higher in the infected group than in the uninfected group. At a cutoff level of PSP 12.96 ng/mL, the sensitivity was 96.2%, the specificity was 88.5%, positive predictive value was 95.8%, negative predictive value was 89.3%, and area under the curve was 0.87. A significant positive correlation between CRP and PSP was found in infected group.* Conclusion*. The high negative predictive value of PSP (89.3%) indicates that the serum PSP level is a good marker for diagnosis of early onset neonatal sepsis and can be used to limit hospital stay and antibiotic use in neonates treated for suspected sepsis.

## 1. Introduction

Neonatal sepsis is a systemic inflammatory response syndrome in the presence of suspected or proven infection of an infant who is 28 days old or younger [1]. Neonatal sepsis remains one of the main causes of mortality and morbidity despite the progress in hygiene, introduction of new and potent antimicrobial agents for treatment, and advanced measures for diagnosis [2, 3]. The incidence of neonatal sepsis varies from 1 to 4 per 1000 live births in developed countries. In developing countries, the incidence varies from 10 to 20/1000 live births [4] and approximately 1% die due to sepsis related causes [5].

Early onset neonatal sepsis (EOS) is bacteremia or bacterial meningitis occurring at 72 h in infants hospitalized in the Neonatal Intensive Care Unit (NICU) [6, 7].

The microorganisms most commonly associated with EOS include* group B Streptococcus (GBS)*,* Escherichia coli (E. coli)*,* Haemophilus influenzae*, and* Listeria monocytogenes* [8]. The main risk factors for EOS include prematurity, low birth weight, febrile illness in the mother within 2 weeks of delivery, foul smelling and/or meconium stained liquor, premature rupture of membranes, prolonged labor, and perinatal asphyxia [9].

Early sepsis warning signs and symptoms are often subtle and can easily be confused with noninfective causes such as apnea, hypothermia, tachypnea, grunting, lethargy, and vomiting. So hematological and biochemical markers have been proposed as being useful indicators for early identification and treatment of septic infants [10]. Moreover, they are used to avoid overtreatment in nonseptic infants to minimize colonization with drug-resistant microorganisms and superinfection with other pathogens [11].

Pancreatic stone protein (PSP)/regenerating protein 1-alpha (Reg), PSP/Reg, and lithostathine are different names for an identical 16 kDa polypeptide belonging to the family of lectin-binding proteins. PSP appears to have protective functions by promoting cellular proliferative responses during beta-cell regenerative processes and epithelial repair. The presence of PSP/reg in peripheral blood during inflammation or after trauma might point toward a specific regulatory response as seen in acute-phase proteins that could lead to activation of immune cells [12]. PSP has been discovered as a sepsis marker in adults, with higher PSP levels predicting sepsis, sepsis-associated multiple-organ failure, and mortality. However, few data are available about its role in neonatal sepsis [13].

## 2. Objective 

The aim of our study was to evaluate the role of pancreatic stone protein as a marker for early onset neonatal sepsis.

## 3. Subjects and Methods

This was a hospital-based prospective study conducted on 104 newborn infants delivered in the Obstetric Ward and admitted to the Neonatal Intensive Care Unit (NICU) of Zagazig University hospitals from April 2014 to April 2015, fulfilling the following inclusion and exclusion criteria.

Inclusion criteria are neonates born after 34 weeks who were admitted within the first 72 h of life to the NICU with suspicion of sepsis. Exclusion criteria are neonates with history of prenatal, natal, and postnatal asphyxia, traumatic tissue injury, congenital anomalies, and metabolic liver disease. Written informed consent was obtained from the parents of the neonates involved in the study as recommended by the Institutional Ethical Committee of Zagazig University and in accordance with the Declaration of Helsinki after full explanation of the purpose and nature of all procedures used.

All newborns were subjected to the following:

(1) Full prenatal, natal, and postnatal history taking with focus on maternal risk factors of sepsis as prolonged rupture of membranes >18 h, chorioamnionitis (foul smelling amniotic fluid), and GBS positivity.
(2) Meticulous neonatal assessment including determination of gestational age and anthropometric measurements including birth weight.
(3) Complete physical examination including neurological, chest, cardiovascular, and abdominal examination together with assessment of clinical manifestations of neonatal sepsis such as temperature instability (<36.5°C or >37.5°C), poor skin perfusion (capillary refill > 3 seconds), poor activity and crying, poor suckling reflex, poor Moro's reflex, pallor, lethargy or irritability, respiratory distress/apnea, tachycardia/bradycardia, arterial hypotension/poor perfusion, seizures/irritability, abdominal distention (ileus), and vomiting.
(4) Routine laboratory investigations including complete blood counts (CBC) which were performed on Sysmex-KX-21 (Sysmex Corporation, Japan) and C-reactive protein (CRP) which was determined using the Tina-quant C-Reactive Protein Gen.3 assays (Roche Diagnostics, Indianapolis, IN) on Roche Modular P800 system. CRP levels below the detection limit were set at 1 mg/L for the analyses.
(5) Blood cultures which were done using brain-heart infusion broth media (Mast Diagnostic DM 106, UK) and were used for primary isolation of the organisms by adding 1 : 10 (v/v) blood to the media according to the recommendations of the WHO. The blood culture bottle and the incubated plates were incubated at 37°C.
(6) Pancreatic stone protein which was measured using a double-antibody sandwich enzyme-linked immunosorbent assay (ELISA) kits (MyBiosource/MBS285689, San Diego, California, USA). Peripheral venous blood samples were centrifuged directly after sampling for 6 min at 3,000 ×g, and the serum obtained was immediately frozen in sterile tubes at −80°C.

The likelihood of infection was assessed at 24–72 h after admission into two categories based on perinatal sepsis risk factors, clinical signs of sepsis, results of conventional laboratory tests (WBC < 10,000 or >26,000/*μ*L), immature leukocyte count >10%, platelet count <150,000/*μ*L, CRP (>5 mg/L), and culture results [14]:

1. The first group is the infected group (52 neonates) which was subdivided into two subgroups according to blood culture results, proven infection (positive blood cultures), and probable infection (negative cultures, ≥3 abnormal laboratory findings).
2. The second group is the uninfected group including 52 neonates (negative cultures, ≤2 abnormal laboratory findings).

### 3.1. Statistical Analysis

Analysis of data was done by Statistical Package for Social Sciences version 19 (Chicago, IL, USA). Qualitative data were represented as frequencies and relative percentages. Chi-square test was used to calculate difference between qualitative variables. Quantitative variables were described as mean, SD, and range. Independent *t*-test was used to calculate difference between quantitative variables in two groups. For nonparametric data, Mann-Whitney *U* test was used to compare quantitative variables between two groups. Pearson's correlation coefficient was used to calculate correlation between quantitative variables. PSP's role in predicting infection was calculated using the following:

 Sensitivity (percent of positives detected correctly identified) = true positives/(true positive + false negative).
 Specificity (percent of negatives detected correctly identified) = true negatives/(true negative + false positives).
 PV+ = true positive/(true positive + false positive), PV− = true negatives/(true negatives + false negatives). Area under the curve (AUC) was derived from the receiver operating characteristic (ROC) curve [15]. *p* value > 0.05 was insignificant, *p* < 0.05 was significant, and *p* < 0.001 was highly significant.

## 4. Results

Our study included 104 neonates (56 males and 48 females); their age ranged from 12 to 72 hours (median: 29 hours), their gestational age ranged from 34 to 40 weeks (mean: 36.63 ± 1.99 weeks), and their body weight ranged from 1 to 4 Kg with mean of 2.25 ± 0.77 Kg.

More than half of the studied neonates were delivered by cesarean section (53.8%); also 28.8% of them had history of PROM, 19.2% of them had history of maternal GBS, 19.2% of them had history of maternal fever, 23.1% of them had history of intrapartum antibiotics, 28.8% of them had history of fetal tachycardia, and 46.2% of them had other risk factors like mother age <18 or >37, positive consanguinity, oligohydramnios, and diabetes mellitus.

In our study, the most common clinical finding among the infected group was weak suckling (92.3%), weak Moro reflex (77%), respiratory distress (69.2%), lethargy (69.2%), and feeding intolerance (65.9%).

In total, 50% infants were classified as infected including 34 neonates (32.7%) with proven infection (positive blood cultures) and 18 neonates (17.3%) classified as probable infection (negative blood cultures), in contrast to 50% infants with unlikely infection. The identified bacteria in the blood cultures included* Staphylococcus aureus*, 12 (23.1%),* Streptococcus agalactiae*, 8 (15.4%),* E. coli*, 8 (15.4%), and* Klebsiella pneumoniae*, 6 (11.5%).

Our data showed no significant difference between uninfected group and infected group regarding the postnatal age at study entry (30 ± 19.27 hrs versus 28.62 ± 16.99 hrs, resp., with *p* = 0.79), gestational age (36.50 ± 2.2 weeks versus 36.77 ± 1.8 weeks, resp., with *p* = 0.63). Also, there was no significant difference in sex distribution between the two groups but there was predominance of male gender in both groups as male and female percentages were 53.8% and 46.2%, respectively. However, we found that there was a significant difference between uninfected group (2.49 ± 0.78) and infected group (2.0 ± 0.69) in body weight (Kg) with *p* = 0.02.  Table 1 shows other perinatal risk factors and Table 2 shows the laboratory finding.

We found significant difference between proven infection and probable infection subgroups as regards the CRP levels (20.1 ± 3.9 versus 6.5 ± 2.1 mg/L, resp., with *p* < 0.001) and PSP levels (34.6 ± 11.6 versus 17.9 ± 2.1 ng/mL, resp., with *p* = 0.019).

As regards the relation between sex and PSP level of the two studied groups, we found that there was a statistically significant difference between males (21.551 ± 10.349 ng/mL) and females (37.456 ± 20.955 ng/mL) of the infected group in PSP level with *p* = 0.02. But there was no statistically significant difference between males (14.904 ± 3.4474 ng/mL) and females (14.006 ± 3.035 ng/mL) of the uninfected group in PSP level with *p* = 0.49. Moreover, no correlation was established between risk factors (mode of delivery, PROM, maternal GBS, maternal fever, intrapartum antibiotics, and fetal tachycardia) and PSP level of the two studied groups.

**Figure 1:**
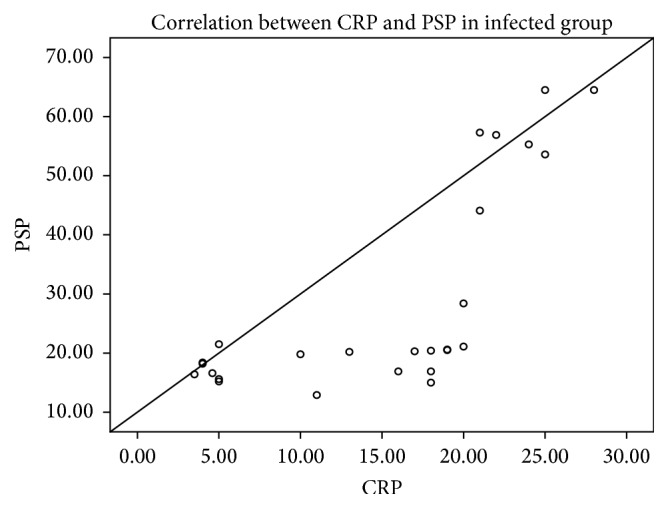
Positive correlation between CRP and PSP of the infected group.

A significant inverse correlation (*r* = −0.47; *p* = 0.02) was found between PSP and body weight in infected group and a highly significant direct correlation (*r* = 0.61; *p* = 0.001) was found between postnatal age at entry of study (hrs) and PSP level in infected group. Also, a significant positive correlation (*r* = 0.73; *p* = 0.000) between CRP and PSP was found in the infected group as shown in Figure 1. Moreover, no correlation was established between other laboratory findings (hemoglobin concentration, hematocrit value, lymphocyte, monocyte, basophil count, and eosinophil count) and PSP level of the two studied groups.

**Figure 2:**
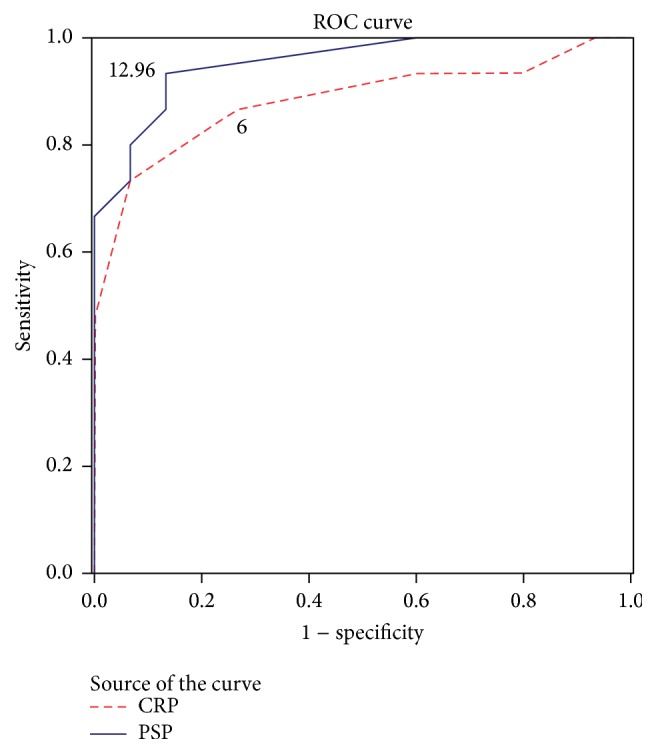
Receiver operating characteristic (ROC) curve evaluating the accuracy of PSP and CRP to distinguish neonatal sepsis. A ROC curve identified that a PSP level (cutoff value) of 12.96 ng/mL has discriminative power between infected and uninfected neonates with 96.2% sensitivity and 88.5% specificity with area under curve of 0.87, while CRP level (cutoff value) of 6 mg/L has discriminative power with sensitivity of 84% and specificity of 65% with area under curve of 0.81.

In this study, at a cutoff level of PSP of 12.96 ng/mL, the sensitivity was 96.2%, the specificity was 88.5%, positive predictive value (PPV) was 95.8%, negative predictive value (NPV) was 89.3%, and the area under curve (AUC) was 0.87 (95% CI: 0.78–0.97; *p* < 0.001), while at a cutoff level of CRP of 6 mg/L, the sensitivity was 84%, the specificity was 65%, positive predictive value (PPV) was 81%, negative predictive value (NPV) was 71%, and the area under curve (AUC) was 0.81 (95% CI: 0.72−0.91; *p* = 0.023), as shown in Figure 2.

Table 1 shows that there was no statistically significant difference between infected group and uninfected group in delivery way or number of patients who had maternal GBS or fetal tachycardia or received intrapartum antibiotics, but there was statistically significant difference between them in PROM and maternal fever.

Table 2 shows that there was no statistically significant difference between infected group and uninfected group in Hb concentration, hematocrit value, lymphocyte, monocyte, basophil count, or eosinophil count. But there was a significant decrease in RBCs, platelets, WBCs, and neutrophil and a highly significant increase in CRP and PSP levels in infected group compared to uninfected group.

## 5. Discussion

Early onset sepsis remains one of the leading causes of neonatal admission. Its early diagnosis presents a clinical dilemma because of the variable and nonspecific clinical presentation [16]. Early diagnosis of sepsis is still difficult and there are no laboratory tests with 100% specificity and sensitivity with the exception of blood cultures which are considered the gold standard for sepsis diagnosis. However, their results need at least 48–72 hours after starting the culture and may be falsely negative if cultures are drawn after antibiotic administration as growth of microorganisms can be suppressed [17]. Hence, a reliable inflammatory marker is required for prompt and accurate identification of neonatal sepsis to avoid delayed or unnecessary treatment [18].

Several studies have reported the significance of procalcitonin in comparison to other traditional sepsis markers in the diagnosis of late onset sepsis [19]. However, the physiological increase of PCT during the first 48 h of life limits the use of PCT in the setting of early onset sepsis [14, 20]. Recent attention has been directed toward the study of the role of new markers as pancreatic stone protein.

We found that 32.7% of the studied groups were provenly infected (positive blood cultures), 17.3% of them were probably infected, and 50% of them were unlikely to be infected. This is similar to other studies which showed lower culture positivity [21]. However, other studies found higher blood culture positivity such as Labib et al. [22] and Mondal et al. [23] who found that blood culture was positive in 74.3% and 61.3% of cases, respectively.

In our study, blood culture results of the infected group agree with other studies which found that 70% of the neonates had positive blood cultures and the identified bacteria, mainly Gram-positive bacteria, included Gram-positive cocci,* Staphylococcus epidermidis*,* S. aureus*,* and Streptococci agalactiae* as the commonest organisms [24]. On the contrary, some studies reported that* Klebsiella* is the commonest isolated organism in septic newborns, with a ratio ranging from 35 to 56% of all isolated organisms [25, 26]. Also, Kuhn et al. [27] reported that* GBS and E. coli* account for most episodes of early onset sepsis in developed countries. These findings may suggest that every neonatal unit has its own pattern of microorganisms, which change from time to time, and antimicrobial combinations should be altered according to culture results. This difference might be attributed to the fact that bacterial profile, resistance, and the use of intrapartum antibiotics differ from one country to another. Presence of Gram-negative organisms in our study may be due to the indiscriminate and inappropriate use of antibiotics, lack of hygienic practices at the place of delivery, poor cord care, and unhygienic newborn care practices.

Our study revealed no significant difference between uninfected group and infected group regarding the gestational age and the postnatal age at study entry. This agrees with other studies [28, 29]. On the other hand, Stoll et al. [30] stated that the risk of early onset sepsis increases with decrease of gestational age because of the inability of white blood cells to carry out phagocytosis, immaturity of the immune system, low complement levels, and hypogammaglobulinemia. We found a statistically significant difference between uninfected and infected groups in body weight (Kg). This agrees with other studies [31]. However, El-Mazary et al.'s [32] study showed that there was no significant difference in body weight between the two groups.

As regards the mode of delivery, we found that there was no significant difference between the two groups. Wilmink et al. [33] found a higher proportion of neonatal sepsis in newborns delivered by elective cesarean section with a gestational age of less than 39 weeks. But Masood et al. [34] found that 74% of neonates who were delivered by spontaneous vaginal delivery (SVD) and 26% of those who were delivered by cesarean section have sepsis. Some environmental factors as unclean environment and unskilled staff are responsible for sepsis in neonates who were delivered by SVD. However, our study revealed a significant difference between two groups as regards premature rupture of membrane and maternal fever, which is consistent with other studies that reported that prolonged PROM and maternal fever were strong predictors of early onset neonatal sepsis [35, 36].

Our study showed a significant decrease in WBCs count in the infected group compared to the uninfected group, which is consistent with other studies [32]. In contrast to our result, Mostafa et al. [37] reported that there was no statistically significant difference in WBCs between septic and control groups; also, Sucilathangam et al. [38] found that total WBCs count was normal in 85% of cases. As total leukocyte count is difficult to be interpreted in the neonatal period because it varies significantly with days of life and gestational age [39], we studied neutrophils count and we found a significant decrease in neutrophils count in the infected group compared to the uninfected group which runs with other studies [40]. Also, Schmutz et al. [41] suggested that neutropenia may be a better marker for neonatal sepsis and has a better specificity than an elevated neutrophil count. Also, we founded a significant decrease in RBCs and platelets count between uninfected and infected groups. This agrees with other studies [23, 29, 32]. Thrombocytopenia may be attributed to bone marrow depression, consumption coagulopathy, platelet sequestration, or a combination of these processes.

Although several new markers of infection have been investigated recently, some studies suggested that CRP is still a significant, sensitive, and specific acute-phase protein for the prediction of sepsis especially in the developing countries [42]. In our study, we found that CRP was significantly higher in the infected group than in the uninfected group which agrees with other studies [23, 37]. In this study, at a cutoff level of CRP of 6 mg/L, the sensitivity was 84% and the specificity was 65%. The lack of specificity was the main disadvantage of CRP; Sucilathangam et al. [38] reported that the sensitivity and specificity of CRP were 50% and 69%, respectively, in culture-proven cases. Also, Schlapbach et al. [28] reported that the sensitivity and specificity of CRP were 36% and 89%, respectively. These values demonstrate that CRP cannot be used as a single marker for the diagnosis of neonatal sepsis. The common causes of false-positive CRP values in neonates are surgery, immunizations, and severe viral infections such as herpes and rotavirus [43].

Our study revealed a significant difference between uninfected and infected infants regarding PSP level which runs with Schlapbach et al.'s [28] study which found that the level of PSP was significantly higher in infected infants (median: 11.3 ng/mL) than in uninfected infants (median: 7.5 ng/mL). In our study PSP levels were significantly higher in the proven infection subgroup than in the probable infection subgroup (34.6 ± 11.6 versus 17.9 ± 2.1 ng/mL, resp., with *p* = 0.019). So, PSP can be used as a biomarker to identify septic patients.

A significant inverse correlation was found between PSP and body weight in infected group as neonates with low birth weight have decreased immunity. This disagrees with Schlapbach et al. [28] who reported a direct correlation between PSP and body weight. Also, we found a highly significant positive correlation between CRP and PSP in the infected group which agrees with Schlapbach et al. [28].

In this study, at a cutoff level of PSP of 12.96 ng/mL, the sensitivity was 96.2%, the specificity was 88.5%, positive predictive value (PPV) was 95.8%, negative predictive value (NPV) was 89.3%, and the area under curve (AUC) was 0.87 (0.78–0.97). Schlapbach et al. [28] stated that a cutoff level of PSP of greater than 9 ng/mL resulted in a sensitivity of 79%, a specificity of 62%, PPV of 39% with NPV of 90%, and the area under curve (AUC) of 0.69 (0.59–0.80). Our results were higher than Schlapbach et al.'s results regarding the sensitivity, specificity, and PPV and were nearly the same regarding NPV because we took a higher cutoff level of PSP (12.96 ng/mL versus 9 ng/mL). At this cutoff level, we found a high NPV to rule out sepsis in the uninfected group and at the same time a high PPV to confirm sepsis diagnosis in the infected group because our sample neonates are product of a different population that has a part of subclinical infection due to inappropriate antenatal care, lack of hygienic practices at the place of delivery, poor cord care, and unhygienic newborn care practices.

The PSP has a rapid laboratory test (<1.3 h) and requires minimal blood volume (<50 *μ*L). Also, PSP is more sensitive and specific and has a good negative predictive value compared to CRP confirming its value as marker to rule out early onset neonatal sepsis.

A limitation of this study is the low incidence of culture-proven EOS (32.7%). The observed low incidence of bacteremia runs with previous studies which showed that culture-proven EOS represents a small percent of the total burden of EOS [27]. Neonatal exposure to maternal antibiotic treatment during labor increases the incidence of false negative blood cultures. Another limitation is inability to use other inflammatory markers as IL-6 and TNF-alpha besides CRP in comparison to PSP for early sepsis diagnosis.

## 6. Conclusion

The high negative predictive value of PSP (89.3%) may allow negative PSP on presentation to rule out sepsis and limit hospital stay and antibiotic use in neonates treated for suspected sepsis. The current study revealed highly significant increase in serum PSP concentrations in the infected group compared with uninfected group, indicating that the serum PSP level is a good marker for diagnosis of early onset neonatal sepsis.

## Figures and Tables

**Table 1:** Risk factors and clinical data of the two studied groups.

Variable	Uninfected group (*n* = 52)		Infected group (*n* = 52)		*χ* ^2^	*p*
	Number	%	Number	%		
*Delivery*						
NVD	11	42.3	11	42.3	0	1
CS	15	57.7	15	57.7		NS

*PROM*						
No	22	84.6	15	57.7	4.59	0.03^*∗*^
Yes	4	15.4	11	42.3		

*Maternal GBS*						
No	23	88.5	19	73.1	1.98	0.16
Yes	3	11.5	7	26.9		NS

*Maternal fever*						
No	24	92.3	18	69.2	4.46	0.03^*∗*^
Yes	2	7.7	8	30.8		

*Intrapartum antibiotics*						
No	20	76.9	20	76.9	0	1
Yes	6	23.1	6	23.1		NS

*Fetal tachycardia*						
No	21	80.8	16	61.5	2.34	0.13
Yes	5	19.2	10	38.5		NS

*Other risk factors*						
No	13	50	15	57.7	0.31	0.58
Yes	13	50	11	42.3		NS
Mother age <18–>37	2		0			
Consanguinity	3		3			
Oligohydramnios	2		1			
Difficult labor	0		2			
Hemorrhage	0		2			
abortion	0		1			
DM	2		0			
HPT	4		1			
UTI	0		1			

*χ* ^2^: Chi-square test.

*∗* means *p* is significant (*p* < 0.05 was significant).

**Table 2:** Lab. finding of the two studied groups.

Variable	Uninfected group (*n* = 52)	Infected group (*n* = 52)	*t*	*p*
*RBCs*				
Mean ± SD	4.75 ± 0.77	4.13 ± 0.95	2.57	0.01^*∗*^
Range	3.2–6	2.3–5.8		

*Hb*				
Mean ± SD	15.69 ± 2.68	14.23 ± 2.86	1.91	0.06
Range	11.5–20.8	8.3–20.7		NS

*HV*				
Mean ± SD	45.11 ± 8.8	40.33 ± 6.51	2.23	0.03
Range	32.7–64.3	24.8–55		

*Platelets*				
Mean ± SD	250.08 ± 67.93	181.5 ± 74.37	3.47	0.001^*∗∗*^
Range	118–388	96–370		

*WBCs*				
Mean ± SD	16.83 ± 4.88	12.35 ± 7.52	2.55	0.01^*∗*^
Range	8.6–27	4.3–31		

*Neutrophil*				
Mean ± SD	7.17 ± 2.63	5.2 ± 4.18	2.09	0.04^*∗*^
Range	3.7–15.2	0.8–24.8		

*Lymphocyte*				
Mean ± SD	7.51 ± 4.31	5.88 ± 3.08	1.58	0.12
Range	0.5–18.8	1.7–15		NS

*Monocyte*				
Mean ± SD	1.49 ± 0.68	1.35 ± 0.95	0.62	0.54
Range	0.34–3.6	0.06–3.8		NS

*Basophil*				
Mean ± SD	0.21 ± 0.21	0.14 ± 0.17	1.35	0.19
Range	0.01–0.8	0.01–0.8		NS

*Eosinophil*				
Mean ± SD	0.49 ± 0.30	0.31 ± 0.62	1.33	0.19
Range	0.06–1.2	0.03–2.8		NS

*CRP (mg/L)*				
Mean ± SD	4.11 ± 2.14	15.23 ± 7.77	7.03	0.000^*∗∗*^
Range	0.5–8	3.5–28		

*PSP (ng/mL)*				
Mean ± SD	14.48 ± 3.23	28.89 ± 17.72	MW	0.000^*∗∗*^
Median	14.2	20.35	4.08	
Range	5.60–21.33	12.92–64.54		

*t*: independent Student's *t*-test. MW: Mann-Whitney test.

*∗* means *p* is significant (*p* < 0.05 was significant).

*∗∗* means *p* is highly significant (*p* < 0.01 was highly significant).
